# Cytokine storm in COVID-19: from viral infection to immune responses, diagnosis and therapy

**DOI:** 10.7150/ijbs.59272

**Published:** 2022-01-01

**Authors:** Yizhou Jiang, Limor Rubin, Tangming Peng, Linlin Liu, Xingan Xing, Philip Lazarovici, Wenhua Zheng

**Affiliations:** 1 Center of Reproduction, Development & Aging, Faculty of Health Sciences, University of Macau, Taipa, Macau SAR, China.; 2 Allergy and Clinical Immunology Unit, Department of Medicine, Hadassah-Hebrew University Medical Center, Jerusalem, Israel.; 3 School of Pharmacy Institute for Drug Research, Faculty of Medicine, The Hebrew University of Jerusalem, Jerusalem 91120, Israel.

**Keywords:** cytokine storm, COVID-19, SARS-CoV-2, immune response, cytokines, diagnosis, therapy, vaccination

## Abstract

The COVID-19 outbreak is emerging as a significant public health challenge. Excessive production of proinflammatory cytokines, also known as cytokine storm, is a severe clinical syndrome known to develop as a complication of infectious or inflammatory diseases. Clinical evidence suggests that the occurrence of cytokine storm in severe acute respiratory syndrome secondary to severe acute respiratory syndrome coronavirus 2 (SARS-CoV-2) infection is closely associated with the rapid deterioration and high mortality of severe cases. In this review, we aim to summarize the mechanism of SARS-CoV-2 infection and the subsequent immunological events related to excessive cytokine production and inflammatory responses associated with ACE2-AngII signaling. An overview of the diagnosis and an update on current therapeutic regimens and vaccinations is also provided.

## 1. Introduction

In the past two decades, before the emergence of COVID-19, coronaviruses were known for two massive pandemics: severe acute respiratory syndrome (SARS) in 2003 and Middle East respiratory syndrome (MERS) in 2012 1-3. The COVID-19 we faced last and this year, is a highly infectious and pathogenic epidemic caused by severe acute respiratory syndrome coronavirus 2 (SARS-CoV-2) infection 4. Despite quarantine measures taken, the virus spread rapidly worldwide without specific effective therapies, but the pandemic has been halted thanks to up-to-date vaccines in countries with national vaccination programs, e.g., Israel 5. By June 2021, SARS-CoV-2 has caused more than 170 million infections and more than 3.7 million deaths worldwide 6. Although most COVID-19 patients have mild symptoms and a good prognosis, some patients may develop serious complications 7. Medical evidence suggests that acute respiratory distress syndrome (ARDS) caused by cytokine storm is a major cause of mortality 8, 9. Cytokine storm is the result of overproduction of cytokines triggered by a variety of infectious and noninfectious diseases or even some therapeutic interventions 10. The excessive cytokines can trigger an uncontrolled immune response, damage lung tissue, and develop into multiorgan failure 10, 11. Therefore, suppression of the cytokine storm is critical for reducing mortality in severe COVID-19 patients. This review summarizes the mechanism of cytokine storm and tissue damage induced by SARS-CoV-2 infection and reviews diagnosis and current therapies with emphasis on vaccination.

## 2. Infection of SARS-CoV-2

SARS-CoV-2 was first isolated from broncho-alveolar lavage fluid of patients in December 2019 12. The virus was then sequenced, and phylogenetic analysis showed that SARS-CoV-2 belongs to the genus *Betacoronavirus* and shares 79% and 50% identity with SARS-CoV and MERS-CoV, respectively 13, 14. Of note, at the whole-genome level, SARS-CoV-2 shares high similarity (nearly 90%) with two bat coronaviruses, bat-SL-CoVZC45 and bat-SL-CoVZXC21, indicating its possible bat origin 13, 14. The sequence identity between SARS-CoV-2 and these two bat coronaviruses is greater than 90% in five gene regions (E, M, 7, N, and 14). However, the S gene and its corresponding S protein were an exception, with approximately 75% and 80% identity, respectively. The S protein is a viral protein that mediates binding to the receptor and fusion with the cell membrane, making it a key determinant of host infection and transmission 15. It can be divided functionally into an N-terminal S1 subunit responsible for receptor recognition and binding, and a membrane-bound C-terminal S2 region responsible for cell membrane fusion 13, 15, both of which are essential for viral entry via the endocytic pathway and infection into host cells 16. The S1 domain is highly variable among different coronaviruses, whereas the S2 domain is more conserved 15, 17. Although the S2 domain of SARS-CoV-2 is identical (approximately 93% similarity) to that of bat-SL-CoVZC45 and bat-SL-CoVZXC21, the S1 domain is quite different (approximately 68% similarity) 13. In addition, the S1 domain of SARS-CoV-2 is similar to that of SARS-CoV, although variations have been found in several key residues, suggesting that they may bind the same receptor 13.

Angiotensin-converting enzyme 2 (ACE2) has been shown to be the cell entry receptor of SARS-CoV 18. Therefore, attempts were made to verify whether it is also the cell entry receptor of SARS-CoV-2. Zhou et al. used SARS-CoV-2 to infect HeLa cells expressing ACE2 protein from humans or other animals, including Chinese horseshoe bats, civet cats, pigs, and mice 14. The results showed significantly efficient virus entry into cells expressing ACE2 except mouse ACE2, whereas cells without ACE2 expression were not infected. In addition to the cell receptor ACE2, other essential cellular proteins such as the transmembrane protease serine 2 (TMPRSS2) and the endosomal cysteine proteases cathepsin B and L (CatB/L), which have S-protein priming, are involved in SARS-CoV infection 19, 20. Hoffmann et al. also found that inhibition of TMPRSS2 activity by camostat mesylate or inhibition of CatB/L by ammonium chloride could only partially block cell entry of SARS-CoV-2 in the presence of the activity of another enzyme 21. Co-treatment of camostat mesylate and E-64d, another CatB/L inhibitor, completely blocked viral infection. These findings suggest that both TMPRSS2 and CatB/L are involved in S-protein priming of SARS-CoV-2. Interestingly, TMPRSS2 appears to have a more dominant role than CatB/L in virus entry, reminiscent of other viral infections. Since camostat is already in clinical use for the treatment of chronic pancreatitis, it is tempting to suggest its potential use for the treatment of COVID-19 disease. Ammonium chloride is also used as an autophagy inhibitor. Since it is still controversial whether and how autophagy is involved in SARS-CoV-2 infection 16, further work is needed to clarify the involvement of autophagy in the intracellular transport of SARS-CoV-2 after its binding and fusion with the target cell membrane.

Binding of the spike protein of SARS-CoV-2 to ACE2 triggers entry of the virus into the cell, but other proteins may also be involved, such as the neuropilin-1 receptor (NRP-1) 22, a transmembrane receptor that lacks a cytosolic protein kinase domain and has high expression in the respiratory and olfactory epithelium. Recent studies have shown that the SARS-CoV-2 spike S1 protein can bind to the b1b2 domain of NRP-1. This interaction occurs via a polybasic amino acid sequence Arg-Arg-Ala-Arg C-terminal sequence on S1 (682RRAR685) that is not conserved in SARS and MERS and is referred to as the "C-end rule" (CendR) motif, which significantly potentiates its entry into cells upon binding to the cell surface receptors neuropilin-1 (NRP-1) and neuropilin-2 (NRP-2) 23. While NRP-1 significantly potentiates SARS-CoV-2 infectivity, an effect blocked by a monoclonal neutralizing antibody to NRP-1, a SARS-CoV-2 mutant with an altered furin' cleavage site was not dependent on NRP-1 for infectivity. Pathological analysis of human COVID-19 autopsies revealed SARS-CoV-2-infected cells, including olfactory neurons positive for NRP-1A in the nasal cavity. In addition, significant upregulation of NRP-1 was found in biological samples from COVID-19 patients compared to healthy controls. Collectively, these data suggest the involvement of NRP-1 in SARS-CoV-2 cell infectivity and define an additional potential target for antiviral intervention in pulmonary and neurological manifestations of COVID-19.

**Figure 1:**
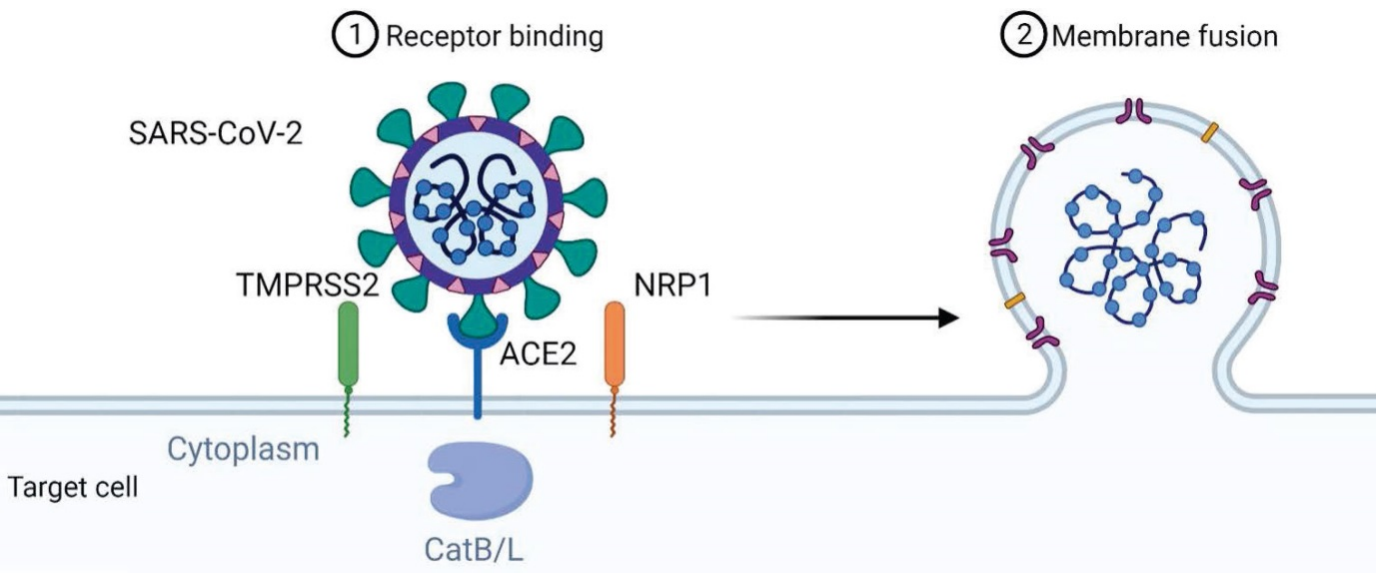
** Mechanism of SARS-CoV-2 viral entry.** SARS-CoV-2 uses angiotensin-converting enzyme 2 (ACE2) as its cellular receptor. The cell entry of SARS-CoV-2 is also dependent on transmembrane protease serine 2 (TMPRSS2), Neuropilin-1 (NRP1) and cathepsin B and L (CatB/L).

The mechanism of viral entry of SARS-CoV-2 via ACE2 could also involve the neutral amino acid transporter B0AT1, also known as SLC6A19. ACE2 may act as a chaperone for membrane trafficking of B0AT1, thereby regulating the uptake of neutral amino acids into intestinal cells 24. A recent study presenting the cryo-electron microscopic structures of ACE2 showed that ACE2 and B0AT1 form a dimer or heterodimer 25. Residues 697 to 716 of the C-terminal segment of ACE2, where proteases such as TMPRSS2 that cleave ACE2 and facilitate viral entry, are involved in dimerization. Therefore, B0AT1 can suppress SARS-CoV-2 infection by blocking the access of TMPRSS2 to the cleavage site on ACE2. It is worth noting that the expression and distribution of B0AT1 is mainly restricted to kidney and intestine, which is lower than that of ACE2 26. Moreover, ACE2 may be able to form homodimers in the absence of B0AT1 because B0AT1 is not involved in dimerization. B0AT1 may play a partial role in the enteric infection of SARS-CoV-2, and this idea should be strengthened by further studies. Figure 1 summarizes the mechanism of viral entry.

## 3. Mechanism of the cytokine storm triggered by SARS-CoV-2 infection

The immune system is vital for the host to fight viral infections. However, excessive immune responses can lead to pathologies 27. Viral entry can trigger a series of immune responses that can further lead to cytokine storm and eventually acute respiratory distress syndrome (ARDS) and death. Both clinical and animal studies have shown that the cytokine storm plays a key role in the pathogenesis of SARS and MERS 28. In clinical studies, higher concentrations of proinflammatory cytokines were found in plasma in critical patients than in mild to moderate patients, suggesting that the cytokine storm is also directly related to the severity of COVID-19 disease 29. The mechanism(s) by which SARS-CoV-2 infection induces cytokine overproduction is not yet fully understood. In the present review, we will mainly focus on the role of ACE2-AngII-related signaling on the cytokine storm induced by SARS-CoV-2 infection.

The ACE2 protein has been found to be most abundant in lung alveolar epithelial cells and small intestinal enterocytes. In addition, ACE2 protein is also highly expressed in endothelial cells and smooth muscle cells of arteries and endothelial cells of veins in all organs studied 30. Analysis of autopsy specimens from SARS patients indicated that SARS-CoV primarily infected epithelial cells of the respiratory tract, consistent with expression levels of ACE2 31. However, despite the lack of ACE2 expression, immune cells, including T lymphocytes, monocytes, and macrophages, were also infected and destroyed by SARS-CoV 30, 31. Furthermore, although SARS-CoV viral particles were detected in other cell types such as epithelial cells of the digestive tract, kidney, and neurons in the brain, many organs with ACE2 expression remained uninfected 30, 31. These discrepancies support the notion that cell entry of SARS-CoV is not solely dependent on ACE2.

As mentioned above, although cell subsets expressing ACE2 are likely targets of SARS-CoV-2, viral entry also depends on other cellular factors such as TMPRSS2. Therefore, cells that co-express ACE2 and TMPRSS2 may be at a higher risk of direct infection with SARS-CoV-2. A recent study that analyzed new and existing single-cell RNA sequencing datasets found co-expressing ACE2+/ TMPRSS2+ cell types, including lung-type pneumocytes II, nasal secretory goblet cells, and ileal absorptive enterocytes, and that ACE2 is a human interferon-stimulated gene (ISG) *in vitro* 32. SARS-CoV-2 has been shown to actively and productively infect cardiomyocytes 33 and myocardial damage correlated with outcome in autopsies showing myocarditis with presence of SARS-CoV-2 viral RNA in hearts from COVID-19 patients 34. Lymphocytopenia is a hallmark of COVID-19 disease 35, 36. Both SARS-CoV and MERS-CoV can infect T cells 31, 37. SARS-CoV-2 has also been found to infect T lymphocytes through S protein-mediated membrane fusion 38. Similar to MERS-CoV, SARS-CoV-2 is unable to replicate in T cells. In addition, T lymphocytes have been reported to be more sensitive to SARS-CoV-2 than to SARS-CoV infection. Because T cells have very low ACE2 mRNA expression, SARS-CoV-2 could infect T cells via a different receptor, such as CD147 39. Extensive T-cell apoptosis was found in COVID-19 patients, in direct correlation with increased expression of pro-apoptotic molecules 40, 41. The question of whether SARS-CoV-2 virus directly causes T-cell apoptosis requires further investigation.

After entry into the host cell, the virus may undergo rapid replication, associated with cell apoptosis, pyroptosis, and production of proinflammatory cytokines and chemokines 42. Most COVID-19 patients were found to have lymphopenia, a decrease in lymphocyte count associated with increased mortality 35, 36. It was hypothesized that lymphopenia is induced by several factors: **i.** Lymphocyte infiltration and sequestration in the lungs or other tissues such as the gastrointestinal tract and lymphoid tissues; **ii**. SARS-CoV-2 direct infection of lymphocytes, as some lymphocytes express ACE2 and SARS-CoV-2 could also infect lymphocytes through other pathways; **iii.** Excess production of pro-inflammatory cytokines including IL-6, which may contribute to additional lymphocyte reduction 35,38, 43. Moreover, the excess pro-inflammatory cytokines and chemokines could further attract neutrophils and monocytes, leading to excessive infiltration of inflammatory cells enhancing the lung injury 27. Indeed, biopsy and autopsy studies have shown lung damage with infiltration of inflammatory immune cells, such as lymphocytes and macrophages, into the lungs of COVID-19 patients 44, 45.

ACE2 functions as a negative regulator of the renin-angiotensin system, which is important for blood pressure regulation and maintenance of fluid and electrolyte balance 46-49. ACE2 balances the activity of angiotensin-converting enzyme (ACE), the homolog of ACE2, and inhibits the production of angiotensin II (AngII) 50. In addition to its cardiovascular modulatory effects, AngII is considered a proinflammatory cytokine 51, 52. AngII activates nuclear factor-κB (NF-κB) through its action on both AngII receptors AT1 and AT2 and mediates inflammatory responses by stimulating the expression of proinflammatory cytokines, chemokines, and adhesion molecules 51, 52. In addition, the renin-angiotensin system plays a critical role in severe acute respiratory failure, including SARS-CoV-2 infection. This conclusion is supported by studies showing that acid aspiration causes reduced ACE2 expression and increased AngII levels, and ACE2 knockout mice developed increased lung failure in the acute lung injury model, compared with wild-type mice 53. Moreover, injection of human recombinant ACE2 protein reduced the severity of lung injury caused by acid treatment and ACE2 deficiency 53. Similar results were also obtained in lung injury induced by viral infections. Reduced ACE2 expression in the lung and increased angiotensin levels in serum II were found after H5N1 infection with avian influenza in a mouse model 54. Importantly, SARS-CoV infection and SARS-CoV spike protein caused reduced ACE2 surface expression 50. The lung injury induced by SARS-CoV spike protein is prevented by ACE2 knockout and by inhibition of AngII receptor type 1 (AT1R) by a specific inhibitor. Therefore, ACE2 has a protective role towards AngII-induced inflammatory response during SARS infection and lung injury caused by other factors. These findings strongly suggest that ACE2 downregulation is critical for SARS-CoV infection-induced lung injury.

After binding to their cellular receptors, viruses frequently use endocytosis machinery to invade host cells 55, 56. Therefore, the downregulation of ACE2 at the cell surface after SARS-CoV infection may be due to the internalization of ACE2. Indeed, flow cytometry and immunostaining showed that the receptor-binding domain of the S protein of SARS-CoV was internalized along with ACE2 57. Shedding of the ACE2 ectodomain may be another mechanism responsible for SARS-CoV infection-induced ACE2 downregulation 58. TACE (TNFα-converting enzyme) is a metalloprotease that releases soluble regulatory factors from cells 59. It has been previously reported that TACE (ADAM 17, a disintegrin and metalloprotease 17) is required for phorbol-12-myristate-13-acetate (PMA)-induced ACE2 shedding 59, 60. TNFα and IL-1β, another proinflammatory cytokine, were able to induce ACE2 shedding 61. Similarly, the spike protein of SARS-CoV-induced ACE2 shedding was dependent on TACE, a biological process that directly correlates with TNFα production 58. These cumulative findings suggest an essential role of proinflammatory cytokines, particularly TNFα in SARS-CoV infection-induced-ACE2 shedding. Interestingly, the shed form of ACE2 was catalytically active and could function in the renin-angiotensin system. However, it has not yet been established that ACE2 shedding is a key step in SARS-CoV-induced lung injury 58. Moreover, in addition to its effect on TNFα release and ACE2 shedding, TACE also cleaves other cellular substrates and releases corresponding molecules such as EGFR ligands and soluble IL-6Rα (sIL-6Rα) 62, 63. Both TNFα and EGFR ligands are capable of activating NF-κB, the major regulator of inflammation 64-66. sIL-6Rα can form a complex with IL-6 and activate another important inflammatory regulatory protein, Signal Transducer and Activator of Transcription 3 (STAT3) 63. NF-κB and STAT3 produce a synergistic effect on IL-6 transcription in nonimmune cells, a process described as IL-6 amplification mechanism 63. In addition, SARS-CoV-2 itself can directly activate NF-κB via pattern recognition receptors 64. The IL-6 amplification leads to an overproduction of pro-inflammatory cytokines and chemokines, including IL-6, and eventually results in a cytokine storm with subsequent ARDS 64. Interestingly, AngII may increase the activity of TACE, thereby promoting the release of its negative regulator ACE2 67. Moreover, AngII also induced the internalization and degradation of ACE2 in an AT1R-dependent manner 68. These findings represent a positive feedback mechanism in the renin-angiotensin system. Therefore, ACE2 downregulation by SARS-CoV-2 infection leads to increased AngII signaling, thereby triggering the excessive inflammatory response and lung injury.

**Figure 2:**
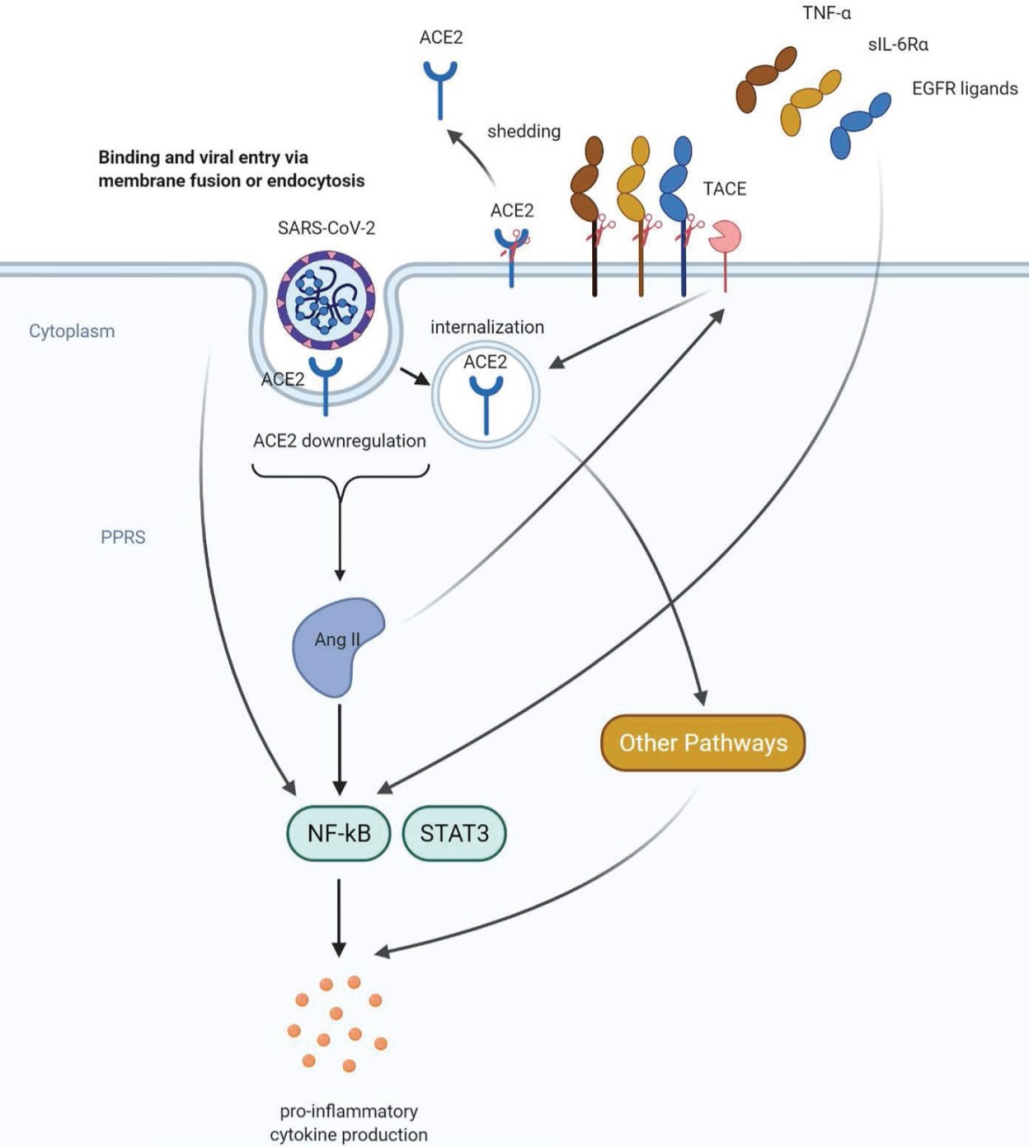
** Proposed mechanisms of COVID-19 -induced cytokine storm**. SARS-CoV-2 can directly activate the key inflammation regulator NF-kB via pattern recognition receptors (PPRs). ACE2 downregulation caused by shedding and internalization of ACE2 leads to increased angiotensin II (AngII) levels and hyperactivation of NF-kB, followed by excessive proinflammatory cytokine production. AngII can stimulate both shedding and internalization of ACE2, representing a positive feedback mechanism in the renin-angiotensin system. In addition, AngII can also promote the shedding of molecules that can activate NF-kB including TNFα, sIL-6Rα and EGFR ligands. These positive feedback mechanisms may further enhance the proinflammatory cytokine production and lead to a cytokine storm.

A recent study investigated the effect of another subset of cytokines (IFN-α2, IFN-γ, IL-4, IL-13, IL-17A, and IL-1β) on ACE2 expression on cultured human primary basal epithelial cells 32. The results showed that IFN-α2 and IFN-γ led to a significant upregulation of ACE2. Since SARS-CoV-2 mediates the downregulation of surface ACE2 protein by several mechanisms including shedding and internalization, IFNs may act in a different way to maintain a certain level of cellular targets to allow the infection of the virus in the host lung. Although the detailed mechanism of each cytokine is not fully elucidated, these findings further imply a tight correlation between cytokine production and ACE2 expression and function. Notably, it was also found that IFN failed to stimulate ACE2 in mice, indicating that the species difference between humans and mice should be considered in modeling COVID-19 disease 32. In addition to functioning in the renin-angiotensin system, ACE2 also interacted with other pathways involved in cytokine production, like the plasma kallikrein-kinin system 69 and Ang-(1-7)/ Mas receptor 70. Moreover, SARS-COV-2 infection could also result in cytokine production by activating complement pathways such as the C3a-C3aR/ C5a-C5aR axis 71. A complex signaling network as proposed in Figure 2, may regulate the COVID-19-associated cytokine storm.

## 4. Clinical outcome and diagnosis of COVID-19 disease

Similar to most viral respiratory illnesses, COVID-19 symptoms include fever and respiratory symptoms such as cough and dyspnea. Additional symptoms may include loss of smell/taste, myalgia, sore throat, rhinorrhea diarrhea, headaches, nausea/vomiting, extreme fatigue, and chest pain 29. COVID-19 infection should be suspected over other respiratory viral infections in the two following scenarios: **i.** Visit/ resident in areas with significant COVID-19 respiratory syndrome outbreaks; **ii.** Close contact (with no use of standard precautions) with a patient who has confirmed/ suspected of COVID-19 infection. Furthermore, the development of dyspnea and exertional desaturation several days after the initial onset of symptoms is a typical symptom 72. Important to mention, approximately 40% of patients infected with SARS-CoV-2 infection are found to be asymptomatic 73. However, they may present with abnormal imaging and laboratory test 74.

Infection with SARS-CoV-2 is classified into three different clinical phases: **i.** Host viral response phase-infection (flu-like symptoms); **ii.** Host inflammatory response/ hypercoagulation phase - ARDS/ elevated acute-phase reaction and cytokines; **iii.** Pulmonary fibrosis formation 75. As mentioned earlier, ARDS is the major life-threatening complication of COVID-19 infection leading to respiratory failure and the need for mechanical ventilation. Additional complications were reported: cardiac arrhythmia, myocarditis, cardiomyopathy 76, thromboembolic (pulmonary embolism**(** 77, deep vein thrombosis (DVT) and stroke 78, secondary infection (bacterial/fungal), an inflammatory complication such as cytokine storm 9, Gullian Barre syndrome 79, Kawasaki disease in children 80 multisystem inflammatory syndrome in adults (MIS-A) 81, hemaphagocytic lymphoistiocytosis (HLH) 82 and toxic shock syndrome. Since the identification of SARS-CoV-2 in humans, a multitude of cardiovascular complications including myocardial injury, heart failure, arrhythmias, thromboembolic disease as well as kidney disease have been reported. Clinical outcomes are worse in patients with COVID-19 with cardiovascular disease and risk factors (eg, hypertension, diabetes, and obesity). Acute cardiac injury, inferred from elevations in cardiac troponin levels, is reported in 8% to 62% of patients hospitalized with COVID-19 and is associated with greater disease severity, including the need for mechanical ventilation, and death 83-85.

The preferred diagnostic assay for SARS-CoV-2 is the nucleic acid amplification test (NAAT) using reverse transcription-polymerase chain reaction to detect SARS Cov-2 RNA in a specimen taken from the upper respiratory tract 86. A single positive test is sufficient for diagnosis, however, if a high clinical suspicion and a negative RT-PCR result, the test should be repeated, lower tract secretion could be examined and a serological test performed. Serological test my also assist in diagnosing asymptomatic patients 87.

## 5. Therapy of COVID-19 disease

As mentioned above, most COVID-19 complications are related to the inflammatory response and cytokine storm, therefore immunosuppression plays an important role in the management of these patients. One of the first medication proposed for COVID-19 therapy was hydroxychloroquine, an antimalarial drug, due to it's *in vitro* evidence of suppressing SARS-CoV-2 replication, in addition to its known immunomodulatory effects 88. Mechanism of action proposed was impairment in the glycosylation terminal of ACE2 and elevation of endosomal pH, resulting in impairment of endosomal mediated viral entrance to the cells 89-91. Hydroxychloroquine effect was assumed to be potentiated by Azithromycin (macrolide antibiotic) administration, leading to a reduction in the viral load 92. Unfortunately, large cohort studies performed showed no significant improvement in clinical outcomes in patients receiving hydroxychloroquine, with/ without Azithromycin treatment 93, 94.

For the present, steroids are the mainstay of treatment. Randomized clinical trials (RCT) presented a decrease in mortality rate among COVID-19 patients aged over 60 years old, who received methylprednisolone therapy 95. RECOVER trial (open label RCT) examined the yield of dexamethasone therapy delivered orally and by intravenous injection. A lower 28-day mortality rate and a shorter hospitalization stay was significantly demonstrated in the treatment subgroup of patients that were in need for oxygen support or mechanical ventilation 96. Interestingly, the combination of methylprednisolone and dexamethasone therapy increased the risk of thromboembolism, despite achieving a rapid anti-inflammatory effect 97. As for the hyper-inflammatory disease phase, biological treatment was in need in order to neutralize the effect of elevated plasma pro-inflammatory cytokines including interleukins (IL-2, 6, 7, 10, gCSF, INFγ, TNFα). Hence, Toclizumab was investigated for this purpose. Toclizumab is a monoclonal antibody that targets membrane bound IL-6 receptor, therefore inhibiting soluble IL-6 signal transduction. At first, reduced mortality was attributed to Tocalizumab treatment in intensive care unit patients 98. However, further studies demonstrated no mortality differences 99 and an increased risk for late onset secondary infections in the treatment group 100. On the other hand, when testing efficacy of low dose Tocalizumab treatment in a subgroup of patients with moderate to severe COVID-19 infection, opposing results were encountered. Low dose Tocalizumab treatment, as add on therapy to standard of care, prevented disease progression in hospitalized patients with moderate COVID-19 infection 101. Patients with severe COVID-19 infection, presenting with elevated levels of inflammatory cytokines (including IL-6), and acute phase reactants, improved lung manifestation in follow-up CT imaging under Tocalizumab treatment and did not progress to develop cytokine release syndrome 102. Recent studies on Tocalizumab treatment conclude lack of efficacy in prevention of intubation and mortality in moderately ill-hospitalized patients 103. Unless presenting with COVID-19 pneumonia, however survival rates remained unchanged, regardless of treatment 104.

In addition to anti - inflammatory agents, the choice of antiviral medication is also under investigation. Favipiravir, an RNA-dependent, RNA polymerase inhibitor prodrug, used as second-line treatment for influenza infection, was proved to demonstrate *in vivo* antiviral activity against SARS-CoV-2 and shorten the time for viral clearance in COVID-19 patients 105. A small RCT demonstrated improved clinical outcome and efficient viral clearance in patients receiving Favipiravir 106 and open-label control study indicated improvement in chest CT findings 107. Favipiravir entered treatment guidelines in several countries such as India and Russia. However, further ongoing clinical trials are in need in order to confirm these results 107.

Remdesivir is another antiviral medication, used on a compassionate-base for patients hospitalized with COVID-19 infection. Remdesivir is a nucleotide analogue prodrug that inhibit RNA polymerase, and showed to have activity against filoviruses (eg. Ebola) 108 and coronaviruses (es. SARS-CoV and MERS-CoV) 105, 109. The use of Remdesivir for hospitalized COVID-19 patients with oxygen saturation of 94 % or less, initially showed improved clinical outcomes 110, with parallel studies presenting contradictory results 111. Further studies indicated superiority to placebo in shortening time to recovery in hospitalized high risk patients that required oxygen support and diagnosed early during illness (>10 days) 112. Remdesivir is an important treatment option and its advantage value in combination with immunomodulators and monoclonal antibodies remains to be studied 113. For example, combination treatment with Baricitinib (JAK inhibitor), accelerated clinical improvement and recovery time in patients receiving high-flow oxygen or noninvasive ventilation 114.

Another aspect of COVID-19 therapy includes prevention of thrombotic micro-and macro-vascular complications with the use of prophylactic or therapeutic anticoagulation therapy. Meta-analysis described the occurrence of VTE (venous thromboembolism) in 31 % of ICU admitted COVID-19 patients despite prophylactic and/or therapeutic anticoagulation, emphasizing the need for personalized dose adjustment and anti-Xa coagulation factor monitoring 115. COVID-19 coagulopathy scoring system is proposed to asses high-risk patients that could benefit from a therapeutic-dose of anticoagulant and perhaps a combination with anti-platelets agents may be beneficial 116. In patients presenting with what appears to be a typical cardiac syndrome, COVID-19 infection should be in the differential diagnosis during the current pandemic, even in the absence of fever or cough. One should have a low threshold to assess for cardiogenic shock in the setting of acute systolic heart failure related to COVID-19. If inotropic support fails in these patients, intra-aortic balloon pump (IABP) device is the first line of mechanical circulatory support, because it requires the least maintenance from medical support staff. When patients on veno-venous extracorporeal membrane oxygenation (VV-ECMO) for respiratory support develop superimposed cardiogenic shock, the addition of an arterial conduit at relatively low blood flow rates may provide the necessary circulatory support without inducing left ventricular distension. Rescue of patients even with profound cardiogenic or mixed shock may be possible with temporary hemodynamic support at centers with availability of such devices. COVID-19 infection can cause decompensation of underlying heart failure, and may lead to mixed shock. Invasive hemodynamic monitoring, if feasible, may be helpful to manage the cardiac component of shock in such cases. Medications that prolong the QT interval are being considered for COVID-19 patients and may require closer monitoring in patients with underlying structural heart disease 117.

As no definite therapy was proved for COVID-19 infection, convalescent plasma therapy is under investigation for rescue therapy in critical ill patients. A 44 % reduction in mortality rate in COVID ‐19 patients receiving convalescent plasma was demonstrated, in addition to improvement in viral clearance. However, it is important to emphasize reservation on this data which is of low quality and indicated high variability among different studies 118. It appears that high-titer convalescent plasma is efficient to prevent disease progression when administered early in disease course to older adults with mild disease 119. In addition immunocompromised patients, especially B cell depleted, may benefit from combined therapy with convalescent plasma and prolonged antiviral treatment 120. Following initial therapy with convalescent plasma, monoclonal neutralizing antibody products targeting different epitopes on the spike protein, entered clinical trials for therapeutic and prophylactic use 121. Neutralizing antibodies proved to reduce viral load especially when administered before initiation of the immune response by COVID-19 infection 122, 123.

As the global pandemic expanded, serological testing and research was vital for COVID-19 prevention and for the development of an efficient vaccination. Most immunocompetent patients will develop an immune response after exposure to SARS-CoV-2'infection and serum antibodies will be detected 1-3 weeks post symptoms onset. SARS-CoV-2 antibodies target two major antigens: Spike glycoprotein (antigen S) present on the viral envelope and nucleocapsid phosphoprotein (antigen N) which interacts with RNA. Different antibody assays are used worldwide that can be classified as binding and neutralizing antibody assays. Binding antibodies are IgA, IgM, IgG type antibodies that recognize SARS-CoV-2 antigens and their expression levels can be measured in serum/ plasma/ saliva, using ELISA method. Neutralizing antibodies are detected in functional laboratory tests analyzing the ability of antibodies to neutralize the virus by incubating live SARS-CoV-2 virus/ recombinant virus with patient serum/ plasma. A strong positive correlation was found between IgG binding antibodies level and neutralizing antibodies level 124. Furthermore, neutralizing antibodies titer was directly correlated to disease severity 125. However, SARS-CoV-2 antibodies levels in the serum decline overtime, with a presumed half-life of 36 days 126. Adaptive immunity essential for efficient vaccine development and T cell response (CD4 and CD8) was recognized, and was directly correlated to anti-SARS-CoV-2 immunoglobulins level 127.

On January 2020 the genetic sequence of SARS-CoV-2 become available. This data accelerated vaccine development. Vaccination development employed different vaccines platforms: live attenuated vaccines, recombinant proteins, virus-like particles, inactivated/ activated vector vaccines, and new platforms of DNA and RNA vaccines. These products are under different developments phases from preclinical research to clinical trials and distribution, following FDA licensing 128. As of June 2021, there are 287 candidate vaccines, 102 undergoing clinical phase and 185 in pre-clinical phase 129. Initially, two major vaccines were under clinical investigation, both targeting SARS-CoV-2 antigen S. The ChAdOx1 nCoV-19 AstraZeneca viral (adenovirus) vector vaccine, expected to generate neutralizing antibodies towards SARS-CoV-2 in 91 % of patients after the first dose, and 100 % after the second dose and elicited a profound T cell response with an acceptable safety profile 130. The other, Moderna vaccine based on mRNA-1273, achieved 50 % neutralizing antibodies after the first dose and 100 % after the second dose and also presented a satisfying safety profile 131. However, Pfizer-BioNTech BNT162b2 mRNA vaccine encoding SARS-CoV-2 full-length spike protein was the first to receive FDA approval following its phase 3 clinical trials. It indicated that a two-dose regimen caused 95 % efficacy in disease prevention and a good safety profile in adults patients 132 and adolescents 133. Israel, representing the current leading country with the highest percentage of vaccinated population, initiated a mass vaccination campaign of the BNT162b2 mRNA vaccine and demonstrated high efficacy in prevention of symptomatic COVID-19 and its serious outcomes: hospitalization, severe illness and death 134, 135. Phase 3 clinical trial results of Moderna mRNA-1273 were published, revealing 94 % efficacy in disease prevention with favorable safety profile 136 and antibody persistence for 6 months following the second dose 137. Sputnik V, Gam-COVID-Vac phase 3 clinical trial indicated 91.6 % efficacy 138. In addition, phase 3 clinical trial demonstrated safety and efficacy of single dose Ad26.COV2.S vaccine 139. ChAdOx1 nCoV-19 AstraZeneca vaccine' phase 2-3 trial revealed similar immunogenicity across all age groups following a boost dose, with better tolerance among older adults 140, 141. Reports of thrombosis and thrombocytopenia following ChAdOx1 nCoV-19 AstraZeneca vaccine raised great concern 142, with a proposed mechanism of antibodies to PF4, unrelated to heparin use 143. However, analysis of adverse events reports exhibited similar risk for venous thromboembolism and thrombocytopenia in vaccinated patients in comparison to the general population. However, the prolonged safety profile, dose adjustment in subpopulation of children, and lasting immunity especially in immunocompromised patients, remain to be investigated.

As mentioned above, vaccines demonstrated 70-95 % efficacy in protection against mild to severe COVID-19 symptoms and almost total mortality prevention. However, numerous new variants of SARS-CoV-2 have emerged representing a possible threat to the control of the COVID-19 pandemic. During the process of SARS-CoV-2 RNA replication in the human host, mutations occur, a virus with a single or multiple mutations is referred to as a "variant" of the original virus. These variants could differ by distinct properties such as binding affinity to the cell receptor, change in the replication rate, increased efficacy of transmission and/or virulence. In the early 2020, the first SARS-CoV-2 variant emerged, exhibiting a D614G substitution in the spike protein 144 . Over a period of six months, the D614G mutation replaced the initial SARS-CoV-2 strain and by June 2020, became the dominant form of the virus circulating globally, due to its high replication rate and/or transmission, by assembling a more functional S protein into the virion 145. At present, four major variants are of concern: **i.** B.1.1.7 (known as 20I/ 501Y.V1 or VOC 202012/ 01 or α-variant) first noted in the UK in December 2020, and characterized by 17 non-synonymous mutations, along with 8 S protein mutations and including the D614G mutation; **ii.** B.1.351 (known as 20H/ 501Y.V2 or VOC 202012/ 02 or β-variant) first identified in South Africa in late 2020, that has multiple mutations in the S protein including K417N, E484K, and N501Y; **iii.** P.1 (known as 20J/ 501Y.V3 or γ-variant) initially found in travelers that came to Japan from Brazil, that has multiple mutations within the receptor binding domain K417T, E484K, and N501Y; and **iv.** B.1.617.2 (known as δ-variant) found in India in late 2020, consisting of D111D, G142D, L452R, E484Q, D614G, and P681R mutations in the S protein and containing within the receptor binding domain three mutations including L452R and E484Q, along with P681R in the furin cleavage site. In all these variants, the mutations in the receptor-binding domain of the spike (S) protein were found to be associated with increased reinfection, escaping binding by neutralizing antibodies of the patient and increased affinity for the human ACE2 receptor 146, 147.

Immunity following COVID-19 infection or vaccination is both humoral, B cell depended and cellular, T cell immunity. SARS-CoV-2 S protein consist of multiple epitopes recognized by T cells, resulting in the production of variable antibodies and a diversity in T cell response 148. Hence, host translation of the S protein following mRNA vaccination induce an immunogenic humoral and cellular response that can overcomes some of the pivotal SARS-CoV-2 variants activities. Regarding the therapeutic effects on the mutant D614G variant, studies showed that the Pfizer-BioNTech BNT162b2 vaccine which was based on the original D614 sequence had a 1.7-2.0 decrease in neutralization, making it less effective 149, 150. The Moderna mRNA-1273 vaccine showed similar neutralization when compared to the original SARS-CoV-2 strain 151. The Moderna and Novavax vaccines exhibited only a moderate reduction in neutralization of the SARS-CoV-2 α-variant compared to the original strain 152 and Pfizer-BioNTech and Moderna vaccines showed no change in S477N, but decreased neutralization of E484K SARS-CoV-2 α-variant 146. In addition, Pfizer-BioNtech and Moderna vaccines showed decreased neutralization towards SARS-CoV-2 α, β and δ-variants, while NVX-CoV2373 vaccine showed good neutralization towards SARS-CoV-2 β-variant 146.

It is clearer now, that adaptive mutations of SARS-CoV-2 could alter its pathogenic potential, and at the same time would raise difficulties on drug and vaccine development. From a clinical point of view, the SARS-CoV-2 variants demonstrating high disease transmission and severity including hospitalization and death, characterized by significant decrease in antibody neutralization and decreased effectiveness of diagnosis and treatment, represent a big challenge. Control measures remain the key for prevention of viral transmission, hence reduction in the opportunity to viral mutation and development of variants. This aspect requires increased efforts to control SARS-CoV-2 spread by developing novel diagnostic kits and increasing research to ascertain the efficacy of the different vaccines and treatments against the new variants. Global mass vaccination programs offer a great promise in controlling this historical pandemic. Unfortunately, in countries such as India and Brazil, the COVID-19 outbreak shows new signs of acceleration, despite the fact that long-delayed vaccination efforts finally gaining steam. We anticipate that COVID-19 vaccines will be administered in every country, to overcome this pandemic and to protect and promote human health. Although COVID-19 vaccines proved to be highly effective in disease prevention, a small percentage of the fully immunized population could still be infected with COVID-19 disease. Several hurdles remain with vaccine administration such as immunocompromised patients, diverse ethnicities and children that have not been satisfactorily represented in vaccine trials. COVID-19 vaccine hesitancy has been also an important aspect that physicians encounter due to large amount of false and misleading information published in the social media 153, 154. It is also important to address that lack of vaccination and associated comorbidities that predispose individuals to get infected with the SARS-CoV-2 δ-variant 155.

## 6. Summary

The immune responses triggered upon viral infections are essential for hosts to eliminate the pathogens. However, the infection of SARS-CoV-2 could cause excessive inflammatory responses, including amplified pro-inflammatory cytokine production and tissue damage, resulting in fatal ARDS. In spite of lacking specific anti-SARS-CoV-2 therapy, suppressing the cytokine storm with corticosteroids is a common approach, which greatly improves the survival rate of COVID-19 patients. Existing drugs that could block the cellular pathways of infection and the excessive inflammatory molecular response, could be also considered for off-label therapy of SARS-CoV-2 disease. Recent studies have suggested that SARS-CoV-2 shares similar cell entry mechanism with SARS-CoV. However, SARS-CoV-2 is much more contagious and may infect humans or animals through novel routes 38, 156. Further studies are still needed to fully elucidate the mechanism behind the cytokine storm induced by SARS-CoV-2 infection and thus providing new targets for therapeutic interventions. The high contagiousness and mortality of COVID-19 and the great challenge to induce long term immunity pose an extraordinary threat for the global health. The emergence of vaccines is the most promising control measure in this global pandemic. The emerging variants not only result in increased transmission, morbidity and mortality but also have the ability to evade detection by diagnostic tests, exhibit decreased susceptibility to treatment including antivirals, monoclonal antibodies and convalescent plasma, and possess the ability to cause reinfection in previously recovered and vaccinated individuals. Children and immunocompromised individuals are at increased risk of developing multisystem inflammatory syndrome. The longer the virus propagates the higher are the chances of mutations. Vaccine hesitancy cases are often undercounted and fully vaccinated populations should still practice preventative measures. Future studies are pending regarding the lasting immunity and efficacy with the emergence of resistant COVID-19 variants. Vaccination towards COVID-19 wild type and variants and revaccination to increase the duration of vaccine-induced immunity represents the present cornerstone of the control of the global COVID-19 pandemic.
